# High-Salt Diet Causes Sleep Fragmentation in Young *Drosophila* Through Circadian Rhythm and Dopaminergic Systems

**DOI:** 10.3389/fnins.2019.01271

**Published:** 2019-11-29

**Authors:** Jiayu Xie, Danfeng Wang, Shengan Ling, Guang Yang, Yufeng Yang, Wenfeng Chen

**Affiliations:** ^1^Institute of Life Sciences, College of Biological Science and Engineering, Fuzhou University, Fuzhou, China; ^2^Institute of Applied Ecology, College of Plant Protection, Fujian Agriculture and Forestry University, Fuzhou, China

**Keywords:** high-salt diet, *Drosophila*, sleep, circadian rhythm, dopaminergic system

## Abstract

Salt (sodium chloride) is an essential dietary requirement, but excessive consumption has long-term adverse consequences. A high-salt diet (HSD) increases the risk of chronic diseases such as cardiovascular conditions and diabetes and is also associated with poor sleep quality. Little is known, however, about the neural circuit mechanisms that mediate HSD-induced sleep changes. In this study, we sought to identify the effects of HSD on the sleep and related neural circuit mechanisms of *Drosophila*. Strikingly, we found that HSD causes young *Drosophila* to exhibit a fragmented sleep phenotype similar to that of normal aging individuals. Importantly, we further showed that HSD slightly impairs circadian rhythms and that the HSD-induced sleep changes are dependent on the circadian rhythm system. In addition, we demonstrated that HSD-induced sleep changes are dopaminergic-system dependent. Together, these results provide insight into how elevated salt in the diet can affect sleep quality.

## Introduction

Although a certain amount of salt is necessary for proper muscle, nerve, and heart function, too much salt can have a serious negative impact on health (He and MacGregor, 2009). A high-salt diet (HSD) is associated with chronic diseases in humans and can also cause damage to the nervous system. In China, most people are halophilic, with an average daily salt intake of 14.5 grams, far exceeding the recommended intake (the Chinese government recommendation is <6 grams per day, and the World Health Organization recommendation is <5 grams per day) (Hvistendahl, 2014; Tan et al., 2019; Zhou et al., 2019). In many countries, HSD is a problem (Trieu et al., 2015).

Sleep is a neurologically related physiological behavior that is a ubiquitous phenomenon from lower organisms to mammals. We spend a third of our lives sleeping, but the purpose and mechanism of sleep remain poorly understood (Frank, 2006; Mignot, 2008). Insufficient sleep and insomnia increase the risk of cardiovascular disease, metabolism and mood disorders, and neurodegeneration (Jansen et al., 2019; McAlpine et al., 2019). Sleep is regulated by two major systems: the circadian system and the homeostatic system. How the circadian rhythm system induces sleep has been characterized, however, very little is known about how homeostasis regulates sleep (Sehgal and Mignot, 2011; Mohawk et al., 2012).

Most previous studies examining the effects of HSD have focused on the cardiovascular system, the immune system, and intestinal flora (He and MacGregor, 2009; Frank et al., 2017; Wilck et al., 2017; Xu et al., 2018). HSD also has important effects on physiological homeostasis, and it has become apparent that excess dietary salt has a negative impact on the nervous system and cognition (Faraco et al., 2018). Excessive salt in the diet may also interfere with sleep (Grandner et al., 2014; Murphy et al., 2016).

In recent years, *Drosophila* has become a powerful, genetically tractable model for studying sleep. *Drosophila* and mammalian sleep have the same characteristics (Dubowy and Sehgal, 2017). When sleep is insufficient, important brain processes such as learning and memory are affected, and, when sleep is insufficient over the short term, this effect can be reversed by supplemental sleep. Many genetic and sleep regulatory molecules are conserved across species (Cirelli and Bushey, 2008; Ly et al., 2018). In human sleep studies, the sleep state is determined by behavioral inactivity and the electrophysiology of brain activity; in flies, sleep is defined as inactivity for more than 5 min (Allada and Siegel, 2008).

In this study, we sought to demonstrate how HSD affects sleep, with the goal of providing a scientific basis for public policy decision-making regarding dietary salt recommendations. We found that HSD fragmented sleep in young flies in a circadian rhythm system-dependent manner. We further showed that HSD caused a dopaminergic nervous system disorder in the brain of *Drosophila*. These findings shed light on the mechanisms underlying homeostatic regulation of sleep.

## Materials and Methods

### Fly Stocks and Rearing Conditions

Flies were raised on standard cornmeal-molasses-yeast medium at 25°C unless indicated otherwise. The line *iso*^31^, which is widely used for circadian behavior studies, was used as the wild-type line (Ryder et al., 2004). The following lines were obtained from the Bloomington *Drosophila* Stock Center: *TH*-Gal4 (Stk #8848), UAS-*NaChBac* (Stk #9468), and UAS-*shibire*^ts^ (Stk #44222). The *TH*-KO^Gal4^/TM6B line was kindly provided by Yi Rao (Peking University, Beijing, China) and has been described previously (Deng et al., 2019). The UAS-*kir2.1*, *clock*^out^, *period*^01^, and *ry*^506^ lines were provided by the lab of Yong Zhang (University of Nevada, Reno, NV, United States).

### Sleep, Survival, and Circadian Rhythm Assays

For sleep analysis, flies were fed on sucrose/agar food (5% sucrose and 1% agar diets) with or without sodium chloride (Sigma-Aldrich, catalog no. S1679), potassium chloride (Sigma-Aldrich, catalog no. P9333), or calcium chloride (Sigma-Aldrich, catalog no. C1016). For the L-DOPA treatment assay, flies were loaded on sucrose/agar food containing 1 mg/mL L-DOPA (Sigma-Aldrich, catalog no. D9628). Adult flies were housed in monitor tubes [5(W) × 65(L) mm) with food. Experiments were performed in an incubator. Lights were turned on at Zeitgeber Time 0 (ZT0) (local time 09:00) and off at ZT12 (local time 21:00). Locomotor activity level was monitored using a Trikinetics *Drosophila* Activity Monitoring (DAM) system (Trikinetics, Inc.) at the temperature indicated under a light-dark (LD) cycle for 6 days. Activity records were collected in 1 min bins and analyzed using a Matlab-based signal-processing toolbox (Levine et al., 2002). Standard sleep measures were calculated from day 4 to day 6 as follows: total sleep duration was the count of bins classified as sleep, mean episode duration was the total sleep divided by the number of episodes; and activity while awake was the total beam breaks divided by the number of minutes with activity. For survival analysis, activity records were also collected under the 12L:12D condition with 1 min bins and then analyzed in Microsoft Excel. For circadian rhythm analysis, fly activities were recorded under LD for 3–4 days, followed by 6–7 days in constant darkness (DD) conditions. Autocorrelation analysis was performed to analyze the rhythmicity index (RI) (Levine et al., 2002). Circadian periods were determined by periodogram analysis (Levine et al., 2002). The periodograms recorded 5 days of activity in constant darkness, beginning 24 h after the last light-off transition.

### Feeding Assay

The method used for the feeding assay was adapted from a previous study (Xu et al., 2008). Briefly, new emerging male flies were entrained at 25°C in 12L:12D for 3 days. The flies were then switched from normal food (cornmeal/yeast/molasses/agar) to blue-colored food (sucrose/agar diets containing 1% FD&C Blue No. 1) with or without 1% sodium chloride at ZT0 for another 3 days. On the 4th day, flies were homogenized in phosphate-buffered saline (PBS) buffer at ZT2 and centrifuged at 12,000 *g* for 25 min. The supernatants were transferred to a new tube and centrifuged at 12,000 *g* for 25 min. Absorbance measured at 625 nm of supernatants from flies fed with normal food was subtracted from the absorbance of the supernatant from blue food-fed flies. The net absorbance reflected the amount of food ingested.

### Ion Chromatography Assay

Three-to-five-day-old adult male flies that were fed with or without 1% sodium chloride in sucrose/agar food for 3 days were collected for the ion chromatography assay at ZT2. Thirty flies were homogenized in deionized (DI) water and centrifuged at 12,000 *g* for 25 min. The supernatants were transferred to a new tube and centrifuged at 12,000 *g* for 25 min, and the volume was raised to 10 mL with DI water. Chloride was measured by treating the sample and standard solutions with nitric acid and silver nitrate and comparing their turbidities. The chloride in the samples was separated on a Dionex IonPac AS18-4 μm column set, a high-efficiency anion exchanger designed for isocratic separation of common anions, on a Dionex ICS-1500 HPIC system. Chloride elutes at 4.0 min. Chloride was quantified by comparison to standards prepared by appropriate dilution of a commercially available 100 mg/L standard run on the same column.

### Immunohistochemistry and Confocal Imaging

Three-to-five-day-old adult male flies that were fed with or without 1% sodium chloride in sucrose/agar food for 3 days were collected for brain dissection at ZT2. Brains were fixed in 4% buffered formaldehyde at 25°C for 2 h, washed in PBS (pH 7.4) with 0.2% Triton X-100 (PBT), blocked in 5% goat serum in PBT (PBST) for 30 min at room temperature, and incubated in rabbit anti-TH (Millipore, #AB152) overnight at 4°C. After three 15 min washes with PBT, the brains were incubated with Alexa Fluor^®^ 488-labeled goat anti-rabbit secondary antibody (Life Technologies) overnight at 4°C, rinsed thoroughly and mounted. Images were taken under confocal microscopy (Leica TCS SP5) at an optical section thickness of 1–2 μm and were analyzed with Image J (NIH).

### Quantitative PCR

Three-to-five-day-old adult male flies that were fed with or without 1% sodium chloride in sucrose/agar food for 3 days were collected at ZT15. Real-time quantitative reverse transcription polymerase chain reaction (qPCR) was conducted on total RNAs extracted from 30 male fly heads using RNAiso Plus (TaKaRa, catalog no. 9108) as per the manufacturer’s protocol. cDNA from a reverse-transcription reaction using HiScript III RT SuperMix (Vazyme, catalog no. R323) was used as a starting material. qPCR was performed using RealStar Green Fast Mixture (GenStar, catalog no. A301) on a LightCycler^®^ 96 (Roche). The following mRNAs were quantified using the listed primers designed using FlyPrimerBank^1^ :

*Actin5C*-f: 5′-CAGAGCAAGCGTGGTATCCT-3′
*Actin5C*-r: 5′-CTCATTGTAGAAGGTGTGGTGC-3′
*TH*-f: 5′-GACCACGATGTCCTCATCAAG-3′
*TH*-r: 5′-CCATCAGATTCATGCTGCTGAAG-3′
*Ddc*-f: 5′-GGGTTTGATTCCCTTCTACGC-3′
*Ddc*-r: 5′-CAAATTGTGCTTGTTTCCCACC-3′
*Dop1R2*-f: 5′-GCACCGCCTCCATACTGAATC-3′
*Dop1R2*-r: 5′-CTCATGGGATAGCTGAAGGGA-3′
*Dop1R1*-f: 5′-GTTAGCGATTGCGGATCTCTT-3′
*Dop1R1*-r: 5′-AGGCCACCCAAGTATCACAAA-3′
*Dop2R*-f: 5′-TAGTTGCCATCTCCATAGACAGA-3′
*Dop2R*-r: 5′-CGGCGGCTATTTTTGTGCTTG-3′
*DopEcR*-f: 5′-TCCAACCTCCTCATTATCGCC-3′
*DopEcR*-r: 5′-GCGGGATACACGGAGAAGG-3′
*Tdc1*-f: 5′-GAGTTTCGCAAATACGGCAAG-3′
*Tdc1*-r: 5′-CGTCGGCTGGTAGCAGTTTT-3′
*Tdc2*-f: 5′-CTCGTTCCCCTCTATCCTGGG-3′
*Tdc2*-r: 5′-CCAGTCGAGCACTATGGTCTC-3′
*Tbh*-f: 5′-GAGTGCAGCAAGGACGTTC-3′
*Tbh*-r: 5′-TTGTGTCGGATAAGCGGTTGG-3′
*Oamb*-f: 5′-TTGGCCGTCCTACCCTTCT-3′
*Oamb*-r: 5′-CGGTCCAGTGATATGGCACAC-3′
*Oct*α *2R*-f: 5′-AATGCCACCCTCAATCCGC-3′
*Oct*α *2R*-r: 5′-GAAGCTGCCATTCAGGAAGAA-3′
*Oct*β *1R*-f: 5′-TGGAGGAGTCAGCCTCACAT-3′
*Oct*β *1R*-r: 5′-TTCAACGGATACGGCGATGC-3′
*Oct*β *2R*-f: 5′-ACATCGTTTGGGTGTTCAAGG-3′
*Oct*β *2R*-r: 5′-GCCCAGTTACTTGCACACTAAA-3′
*Oct*β *3R*-f: 5′-AACGTGGCTGACCTTCTGG-3′
*Oct*β *3R*-r: 5′-CTGTTAATGTTGCAGGTTGCAG-3′
*Oct-TyR*-f: 5′-CGGCATTGAGTACGGCTCAG-3′
*Oct-TyR*-r: 5′-CGATAATGACCGAGAGAACCAGG-3′

### Statistical Analysis

All statistical analyses were performed with Prism 7 (GraphPad Software). The Mann–Whitney test was used to compare two columns of data. For multiple comparisons, a Kruskal–Wallis test followed by Dunn’s post test was conducted. Comparison of survival was conducted using the Mantel-Cox log-rank test. Statistical significance is denoted by asterisks: ^∗^*P* < 0.05, ^∗∗^*P* < 0.01, ^∗∗∗^*P* < 0.001, ^****^P < 0.0001.

## Results

### HSD Suppresses Sleep Consolidation in Wild-Type Flies

**FIGURE 1:**
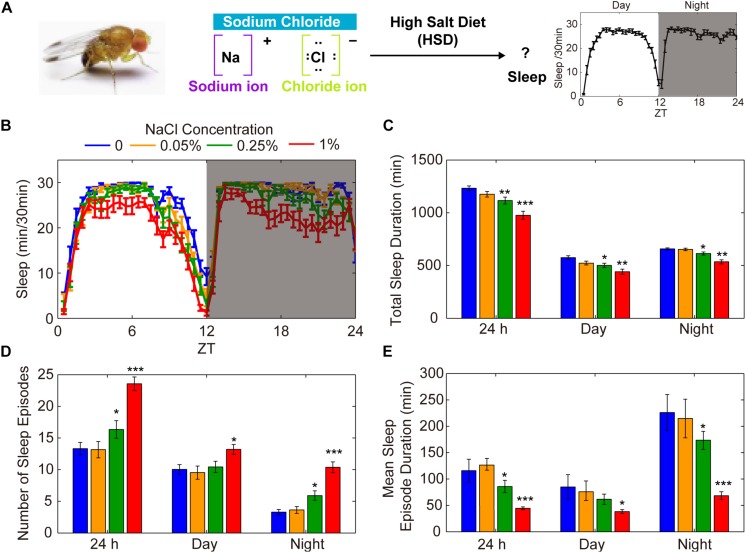
HSD causes fragmented sleep in young male flies. **(A)** HSD-induced changes in sleep were investigated in *Drosophila*. **(B–E)** Two-to-three-day-old male flies (*n* = 48–64 per group) were fed no added NaCl (blue), 0.05% NaCl (yellow), 0.25% NaCl (green), and 1% NaCl (red) and the following were determined over a 24 h period, in daytime, and in nighttime: **(B)** minutes of sleep per 30-minute period, **(C)** average sleep duration, **(D)** number of sleep episodes, and **(E)** mean duration of each sleep episode. Values plotted are means ± S.D.; ^∗^*P* < 0.05; ^∗∗^*P* < 0.01; ^∗∗∗^*P* < 0.001 relative to sucrose/agar diet control.

To explore the possible involvement of HSD in the regulation of *Drosophila* sleep, we investigated the effects of exogenous sodium chloride feeding on fly sleep using the DAM system (Pfeiffenberger et al., 2010; Figure 1A). Three-to-five-day-old wild-type (*iso*^31^) adult males or females were fed sucrose/agar food containing different concentrations of sodium chloride. We found that the total sleep duration decreased in a dose-dependent manner during both the day and the night (Figure 1B). The sleep-inhibiting effect of sodium chloride was more significant for nighttime sleep than daytime sleep, especially in the female flies (Figure 1C and Supplementary Figure S1). The HSD-induced sleep-fragmentation phenotype is demonstrated by a striking increase in the number of sleep episodes and a decrease in the mean sleep episode duration in flies fed high doses of sodium chloride (Figures 1D,E). In contrast, administering sodium chloride did not appear to make flies hyperactive as measured by activity per waking minute throughout the whole day or during the daytime or nighttime (Supplementary Figure S2). Next, we sought to determine how quickly flies change their sleep in response to HSD and whether the sleep fragmentation effects of HSD are reversible by returning dietary salt intake to normal. To this end, after 4 days of normal food or HSD sleep assays, flies were transferred to the opposite foods on the 5th day. We found that flies began to show sleep change responses to altered salt levels on the second day after food transfer (Supplementary Figure S3). Moreover, when flies were transferred from HSD to normal food, it takes at least 2 days for the HSD sleep-fragmentation phenotype to be erased (Supplementary Figure S3). Together, these data show that HSD has inhibitory effects on sleep consolidation in young flies through a build-up of ionic concentrations.

To test whether food with other salts instead of sodium chloride can also affect sleep, we added potassium chloride or calcium chloride to the food and monitored sleep changes. We found that there were no effects on sleep when potassium chloride was added (Supplementary Figure S4A). However, when 1% of calcium chloride was added, it also caused sleep fragmentation (Supplementary Figure S4B). Considering previous studies, it is not surprising to find that the supply of calcium chloride causes sleep changes. Calcium levels in Kenyon cells show a decline when flies fall asleep and increase when they wake up (Bushey et al., 2015). Moreover, it has been shown that the Ca^2+^ channel negatively modulates sleep (Jeong et al., 2015). Thus, these findings suggest that the sodium ion but not the chloride ion has effects on sleep.

### High Salt Supplementation Does Not Alter Food Intake

**FIGURE 2:**
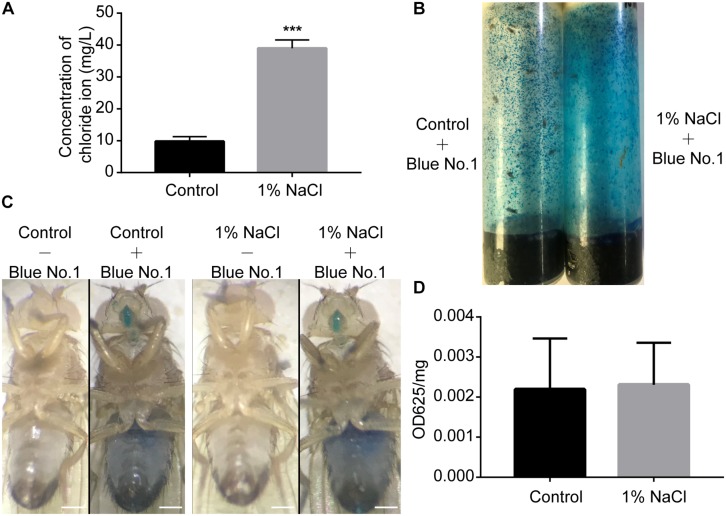
*Drosophila* display normal feeding despite a high concentration of sodium chloride in food. **(A)** Concentration of chloride in flies fed a sucrose/agar diet and a diet with 1% NaCl (*n* = 30 per group). **(B)** Flies in feeding tubes containing blue dye. **(C)** Representative images of flies fed control or 1% NaCl with or without blue dye. Scale bars: 0.2 mm. **(D)** Food consumption of flies fed a control diet or a diet supplemented with 1% NaCl (*n* = 5). Values plotted are means ± S.D.; ^∗∗∗^*P* < 0.001 relative to sucrose/agar diet control.

Akin to mammals, flies may prefer low-salt food and reject high-salt food (Zhang et al., 2013). To test whether salt concentrations influence food consumption by flies, we first performed an ion chromatography assay to detect the concentration of chloride ions in the fly bodies. Groups of male flies were fed sucrose/agar food containing 1% sodium chloride or normal food under 12 h light:12 h dark (LD) cycles for 3 days. After feeding, flies were homogenized at ZT2, and the chloride ion concentrations in the supernatant were determined using a high-pressure ion chromatography assay. Flies fed a diet supplemented with 1% sodium chloride had nearly four times more chloride ions than the control group fed a sucrose/agar diet (Figure 2A). We next used a food dye to evaluate food consumption spectrophotometrically (Xu et al., 2008). Flies were fed dye-labeled food for 3 days (Figures 2B,C). After homogenization at ZT2, the dye was quantified spectrophotometrically. Food consumption was no different for flies fed with 1% sodium chloride than for flies fed the sucrose/agar diet (Figure 2D). These data indicate that the sleep phenotypes resulting from the administration of sodium chloride were not caused by starvation.

### Sleep in Aged Flies Is Not Sensitive to HSD

**FIGURE 3:**
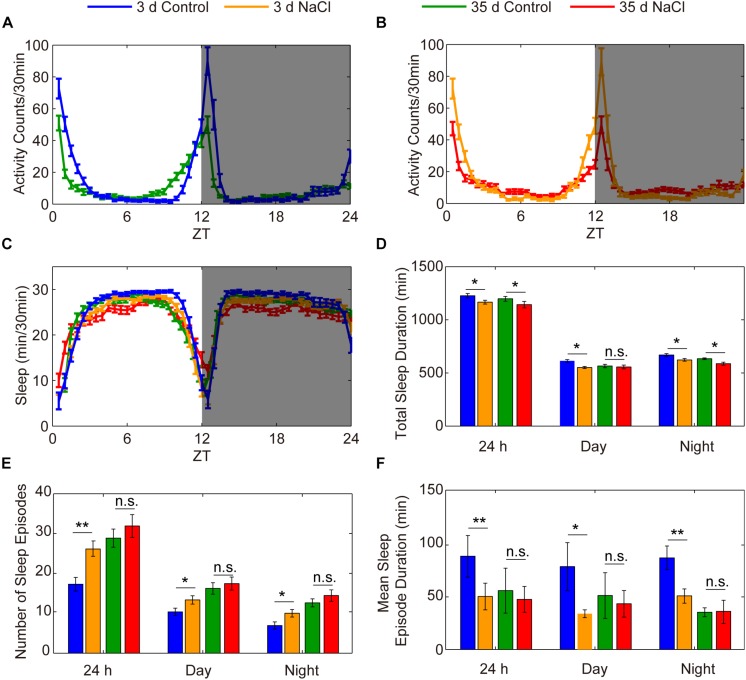
HSD has no effects on the sleep of aged flies. **(A–F)** Three-day-old flies were fed a diet not supplement with sodium chloride (blue), 3-day-old flies were fed a diet supplemented with 1% NaCl (yellow), 35-day-old flies were fed a sucrose/agar diet (green), and 35-day-old flies were fed a diet supplemented with 1% NaCl (red), and the following were evaluated for a 24 h period, in daytime, and in nighttime: **(A)** locomotor activities of flies with sucrose/agar diet, **(B)** locomotor activities of flies with 1% NaCl, **(C)** minutes of sleep per 30-minute period, **(D)** average sleep duration, **(E)** number of sleep episodes, and **(F)** mean sleep episode duration. Values plotted are means ± S.D. ^∗^*P* < 0.05; ^∗∗^*P* < 0.01; n.s.: not significant.

The decrease in sleep duration and sleep consolidation in flies fed a diet high in salt is similar to what has been observed in aged individuals compared to young individuals (Vienne et al., 2016). To assess the effect of HSD on the sleep of aged flies, we used 35-day-old flies to conduct sleep assays. Young and aged flies were fed standard or 1% sodium chloride-containing food, and their sleep was analyzed. We found that the locomotor activities of 35-day-old aged flies with or without HSD were both suppressed compared to young flies (Figures 3A,B). As expected from previous results (Vienne et al., 2016), we found an increase in sleep fragmentation in aged flies compared to young flies (Figures 3C–F). Moreover, we found that young flies fed HSD had significantly decreased sleep duration and increased sleep fragmentation compared to young flies fed the standard diet (Figures 3C–F). However, we did not further find that HSD showed obvious effects on the sleep fragmentation of 35-day-old aged flies (Figures 3C–F). Conversely, HSD shortened the life span of aged flies. All of the 50-day-old aged flies fed 1% sodium chloride-containing sucrose/agar food died within 2 days after initiation of the diet, for an average life expectancy of only 41.07 ± 3.12 h. In contrast, 50-day-old aged flies fed the sucrose/agar diet had an average life expectancy of 235.4 ± 6.54 h after diet initiation (Supplementary Figure S5). HSD in young flies also led to shortened life expectancy in a sodium chloride dose-dependent manner (Supplementary Figure S5). When fed a sucrose/agar diet, the average life expectancy was more than 22.5 ± 0.83 days, whereas flies fed a diet supplemented with 1% sodium chloride from 3 to 5 days of age had an average life expectancy of 8.05 ± 0.57 days (Supplementary Figure S5). In summary, the total sleep decrease and sleep fragmentation increase in young flies as a function of sodium chloride is similar to the effects of aging on sleep, but it is likely that the sleep fragmentation observed under these two conditions is mediated by independent mechanisms.

### HSD-Induced Sleep Changes Are Dependent on the Circadian Rhythm System

**FIGURE 4:**
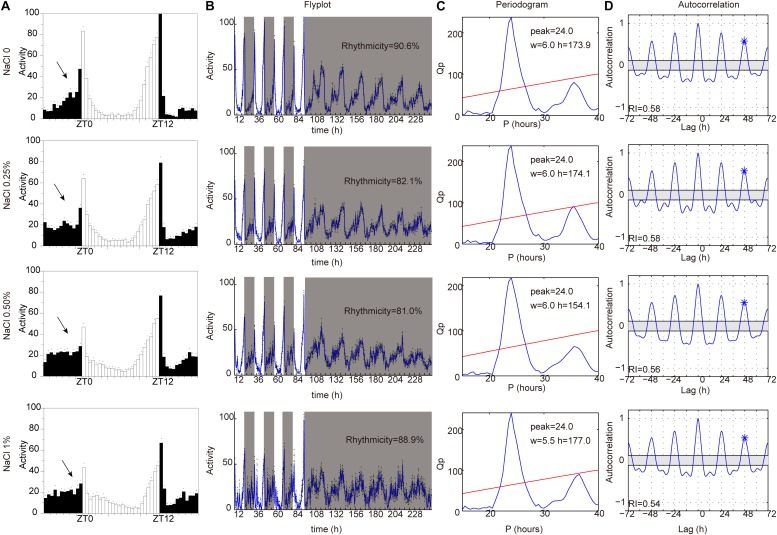
HSD slightly impairs endogenous circadian rhythm. *iso*^31^ adult flies fed from top to bottom with sucrose/agar food and with food supplemented with 0.25% NaCl, 0.5% NaCl, and 1% NaCl. **(A)** Daily locomotor activities were assayed. Data plotted are from LD2-3. White bars represent day; black bars represent night. ZT is indicated on the *x*-axes. The dots above the bars indicate standard error of the mean (SEM). The arrows indicate the morning anticipation peak. **(B)** The temporal distribution of activity, with counts collected every 30 min for several days. Gray shading indicates a period of darkness. Behavioral rhythms were determined by periodogram analysis. Percent of rhythmic flies under DD conditions are shown. **(C)** Periodogram analysis. Amplitude (h) and width (w) of the periodogram peaks are indicated. **(D)** Autocorrelation analysis. The asterisk above the third peak indicates the point used to assess the rhythmicity index (RI).

Sleep is thought to be broadly regulated by two distinct processes: the circadian system controls the timing of sleep, and the homeostatic sleep system controls the duration of sleep (Sehgal and Mignot, 2011; Mohawk et al., 2012). To identify whether HSD affects circadian rhythms, we measured locomotor activity in HSD flies under LD and then under constant darkness (DD). Under LD, flies have bimodal activity patterns that peak around dawn and dusk (Allada and Chung, 2010; Dubowy and Sehgal, 2017). Their locomotor activity gradually increases before the lights-on and lights-off transitions, a phenomenon termed anticipation. In DD conditions, the evening activity peak persists and reoccurs with a period of near 24 h (Allada and Chung, 2010; Dubowy and Sehgal, 2017). Although the morning anticipation was slightly impaired in HSD flies relative to that in control animals, behavioral rhythms under DD conditions were as clearly defined in HSD flies as in controls (Figure 4). Thus, HSD does not have a dramatic impact on circadian rhythms but has strong effects on sleep.

**FIGURE 5:**
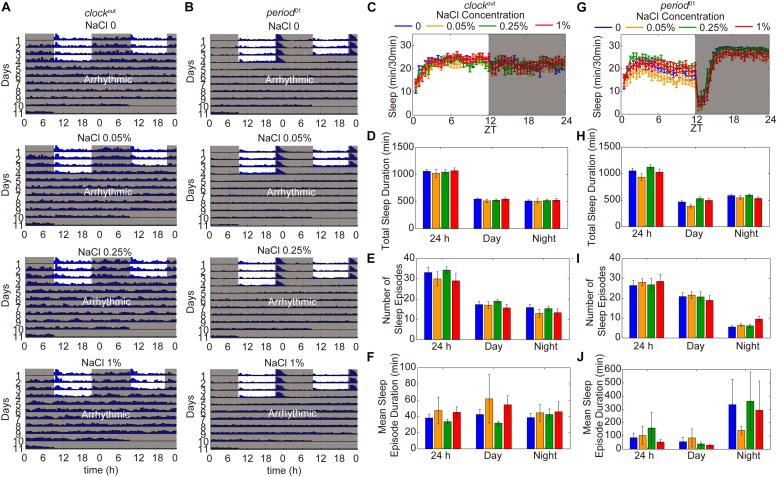
HSD-induced sleep changes are dependent on the circadian rhythm system. **(A)** Actograms of *clock*^out^ and *period*^01^ mutants fed, from top to bottom, sucrose/agar and diets supplemented with 0.05, 0.25, and 1% NaCl. Locomotor activity was monitored in LD conditions for 4 days and then in DD for 7 days. Within each actogram, two consecutive days of activity are shown in each row; the 2nd day is re-plotted in the left half of the next row down, and thus consecutive days of locomotion can be viewed both horizontally and vertically. The heights of bars within a given actogram row reflect varying amounts of locomotion per 30 min data-collection bin. **(B)** Actograms of *period*^01^ mutants fed, from top to bottom, sucrose/agar diet and diets supplemented with 0.05, 0.25, and 1% NaCl. **(C–F)** *clock*^out^ flies (*n* = 40–50 per group) were fed sucrose/agar diet (blue) or a diet supplemented with 0.05% NaCl (yellow), 0.25% NaCl (green), or 1% NaCl (red) and the following were determined for a 24 h period, daytime, and nighttime: **(C)** minutes of sleep per 30-minute period, **(D)** average sleep duration, **(E)** number of sleep episodes, and **(F)** mean sleep episode duration. **(G–J)** *period*^01^ flies (*n* = 45–60 per group) were fed sucrose/agar diet (blue) or a diet supplemented with 0.05% NaCl (yellow), 0.25% NaCl (green), or 1% NaCl (red) and the following were determined: **(G)** minutes of sleep per 30-minute period, **(H)** average sleep duration, **(I)** number of sleep episodes, and **(J)** mean sleep episode duration. Values plotted are means ± S.D.

To identify whether the reduction of sleep duration and sleep consolidation under HSD is circadian rhythm-dependent, two different circadian rhythm-associated mutants, *clock*^out^ and *period*^01^, were analyzed (Konopka and Benzer, 1971; Liu et al., 2017). As shown in previous studies (Konopka and Benzer, 1971; Liu et al., 2017), the *clock*^out^ and *period*^01^ mutants have disrupted activity/rest rhythm compared to wild-type flies and also display arrhythmic activity under DD (Figures 5A,B). When fed a HSD, the sleep patterns of *clock*^out^ and *period*^01^ mutant flies were normal (Figures 5C–J). Given that the *period*^01^ mutant fly we used carries a *ry*^506^ allele on the third chromosome, we also evaluated sleep patterns of *ry*^506^ flies fed different concentrations of sodium chloride. The *ry*^506^ flies responded to HSD with fragmented sleep (Supplementary Figure S6). These findings support the idea that HSD affects the sleep architecture, which requires normal functioning of the circadian rhythm system.

### HSD Elevates Tyrosine Hydroxylase Levels in Dopaminergic Neurons

**FIGURE 6:**
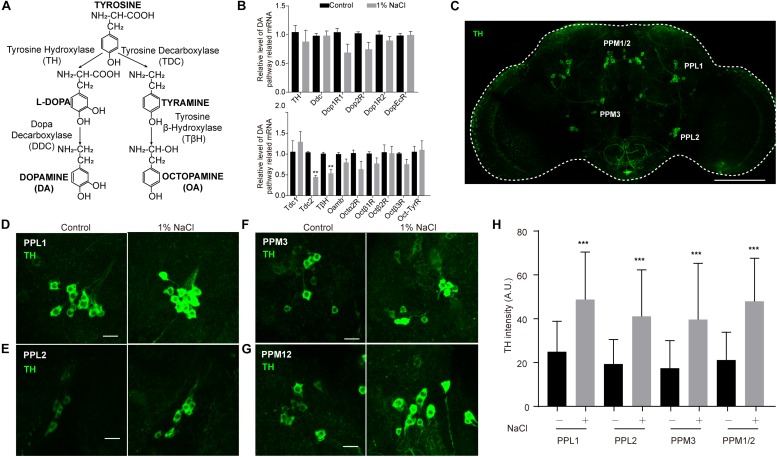
Tyrosine hydroxylase levels are abnormally high in the dopamine neurons of flies fed a high-salt diet. **(A)** DA and OA synthesis pathways in *Drosophila*. **(B)** qPCR analysis of DA and OA synthesis pathway-related genes in the heads of control flies and flies fed a diet supplemented with 1% NaCl. Three independent biological samples were conducted. **(C)** Representative image of the central brain of a control fly stained with anti-TH. The positions of the four major DA cell clusters are indicated. The white outline shows the brain. Scale bar: 100 μm. **(D–G)** Images of PPL1, PPL2, PPM3, and PPM12 cell clusters stained with anti-TH from a control fly and a fly fed with HSD. Scale bars: 10 μm. **(H)** Quantification of TH intensity in the dopamine neurons (*n* = 8–10 per group). Values plotted are means ± S.D.; ^∗∗^*P* < 0.0, ^∗∗∗^*P* < 0.001 relative to levels in the control.

Next, we set out to confirm whether HSD affects sleep through the physiological homeostatic regulation system, specifically the neurotransmitter systems. The monoamine neurotransmitters dopamine (DA) and octopamine (OA) have been implicated in sleep regulation in mammals and in *Drosophila* (Dubowy and Sehgal, 2017). Previous studies suggest a strong wake-promoting role for DA and a sleep-promoting role for OA (Ueno et al., 2012; Deng et al., 2019). DA and OA are both derived from tyrosine (Wright, 1987; Figure 6A). The tyrosine hydroxylase (TH) encoded by the *pale* locus in *Drosophila* is the rate-limiting enzyme for DA synthesis from tyrosine to L-DOPA; L-DOPA is converted to DA by the decarboxylase DDC (Wright, 1987; Yamamoto and Seto, 2014; Figure 6A). In *Drosophila*, four G-protein-coupled DA receptors have been reported: two D1-like receptors (encoded by *Dop1R1* and *Dop1R2*), one D2-like receptor (encoded by *Dop2R*), and one non-canonical receptor (encoded by *DopEcR*) (Yamamoto and Seto, 2014). In parallel, the first step in OA biosynthesis is catalyzed by tyrosine decarboxylases, which convert tyrosine to tyramine; OA is synthesized from tyramine via the action of the enzyme tyramine-β-hydroxylase (*T*β*H*) (Wright, 1987; Cole et al., 2005; Figure 6A). Two *Drosophila* genes, *Tdc1* and *Tdc2*, encode tyrosine decarboxylases. *Tdc1* is expressed non-neuronally, whereas *Tdc2* is expressed neuronally (Cole et al., 2005). Four groups of six OA receptor-encoding genes have been identified: octopamine receptor in mushroom bodies (*OAMB*), α2-adrenergic-like octopamine receptor (*Oct*α*2R*), octopamine β receptor (*Oct*β*1R, Oct*β*2R, Oct*β*3R*), and octopamine-tyramine receptor (*Oct-TyrR*) (Evans and Maqueira, 2005; Hauser et al., 2006; Ohhara et al., 2012; Qi et al., 2017).

To test whether HSD alters the expression of genes that are involved in the DA and OA pathways, we subjected flies to a normal diet or a diet supplemented with 1% sodium chloride and then performed quantitative real-time PCR (qPCR) analysis of total RNA prepared from adult heads. OA pathway-related genes, especially *Tdc2* and *T*β*H*, were downregulated in HSD flies compared to controls (Figure 6B). In contrast, no obvious differences in mRNA levels were found for DA pathway-related genes between HSD flies and controls (Figure 6B).

Because DA neurons make up a small fraction of the neurons in the brain (White et al., 2010), we also tested whether HSD caused changes in levels of proteins involved in DA biosynthesis in these neurons. HSD and control flies were subjected to immunocytochemistry of brain whole mounts after 3 days of feeding with a diet supplemented with 1% sodium chloride. We focused on five major DA cell clusters in the central brain that can be distinguished based on anatomical position: paired posterior lateral 1 and 2 (PPL1 and PPL2), paired posterior medial 1 and 2 (PPM1/2), paired posterior medial 3 (PPM3), and paired anterior medial (PAM) (White et al., 2010; Figure 6C and Supplementary Figure S7). In HSD flies, levels of TH protein in PPL1, PPL2, PPM1/2, and PPM3 were higher than in controls (Figures 6D–H), suggesting that the up-regulated TH levels may be due to post-transcription or post-translation regulation. However, there were no obvious differences in TH levels in PAM neurons from HSD flies compared to controls (Supplementary Figure S7). These data provide evidence that HSD regulates sleep by modulating the DA pathway.

### The Dopamine Pathway Is Involved in HSD-Induced Sleep Changes

**FIGURE 7:**
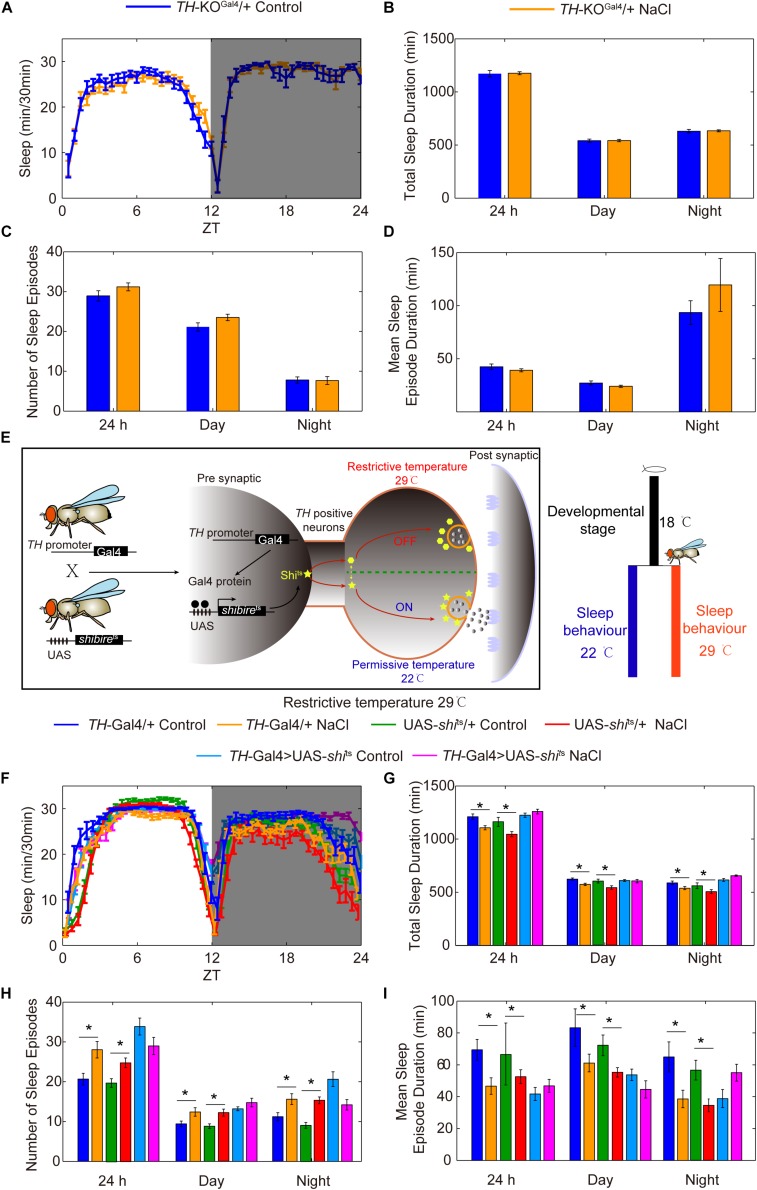
Reduction of the rate-limiting enzyme in DA synthesis or silencing of DA neurons blocks the effects of HSD on sleep changes. **(A–D)** *TH*-KO^Gal4^ heterozygous mutant flies (*n* = 60–80 per group) fed with or without 1% NaCl were monitored, and the following were determined for a 24 h period, in daytime, and in nighttime: **(A)** minutes of sleep per 30-minute period, **(B)** average sleep duration, **(C)** number of sleep episodes, and **(D)** mean sleep episode duration. **(E)** Schematic of the UAS-*shi*^ts^ transgene driven by *TH*-Gal4 used to silence DA neurons. **(F–I)** *TH*-Gal4 > UAS-*shi*^ts^ flies and control flies (*n* = 40–60 per group) were reared at 18°C, and were then transferred to 29°C and fed a sucrose/agar diet or a diet supplemented with 1% NaCl. The following were determined for a 24 h period, in daytime, and in nighttime: **(F)** minutes of sleep per 30-minute period, **(G)** average sleep duration, **(H)** number of sleep episodes, and **(I)** mean sleep episode duration. Values plotted are means ± S.D.; ^∗^*P* < 0.05.

Given that TH is the rate-limiting enzyme for DA synthesis, we investigated whether the DA pathway is involved in HSD-induced sleep regulation by using a *TH* mutant line (Deng et al., 2019). Because *TH*-null mutations are lethal, a heterozygous *TH*-KO^Gal4^ line in which the first coding exon of *TH* has been replaced with *Gal4* was used (Deng et al., 2019). We found that TH protein levels in the heads of *TH* heterozygous mutants were lower by about two-fold than levels in the heads of wild-type flies (Supplementary Figure S8). Groups of *TH* heterozygous mutant flies were then fed either sucrose/agar food or food supplemented with 1% sodium chloride and subjected to sleep analysis. We found that *TH* heterozygous mutant flies had the same sleep patterns whether they were fed a sucrose/agar diet or subjected to HSD (Figures 7A–D), suggesting that HSD induces fragmented sleep in young flies through the dopamine system.

To determine whether DA neurons are important for HSD-induced sleep changes, we utilized the Gal4/UAS system to test whether silencing or activating DA neurons alters sleep changes in response to HSD. GAL4/UAS is a binary system with two parts: the yeast transcription activator protein GAL4 (expressed in a tissue and/or time-specific pattern) activates transgenes under the control of a UAS (upstream activating sequence) promoter activated by GAL4. This system enables spatiotemporal-specific transgene expression (Brand and Perrimon, 1993). Here, a DA neuron-specific expressing driver *TH*-Gal4 was used (Friggi-Grelin et al., 2003; Figure 7E). Possibly due to a critical role of DA-mediated signaling in fly development, inactivation of DA neurons by *TH*-Gal4-driven expression of an inward rectifying potassium channel, UAS-*Kir2.1* (Baines et al., 2001), caused lethality (data not shown). To conditionally suppress DA neurons at the adult stage, we expressed a temperature-sensitive synaptic blocker, UAS-*shibire*^ts^ (*shi*^ts^), under the control of *TH*-Gal4. The *shi*^ts^ allele is defective in synaptic vesicle recycling at temperatures above 29°C; above this temperature, there is rapid and reversible inhibition of synaptic transmission (Koenig et al., 1983; Figure 7E). Upon a shift from 18°C to restrictive temperature 29°C after eclosion, the sleep fragmentation responses to HSD disappeared in *TH*-Gal4 > UAS-*shi*^ts^ flies but not in control flies (Figures 7F–I), while the flies under permissive temperature 22°C showed sleep changes due to HSD (Supplementary Figure S9).

**FIGURE 8:**
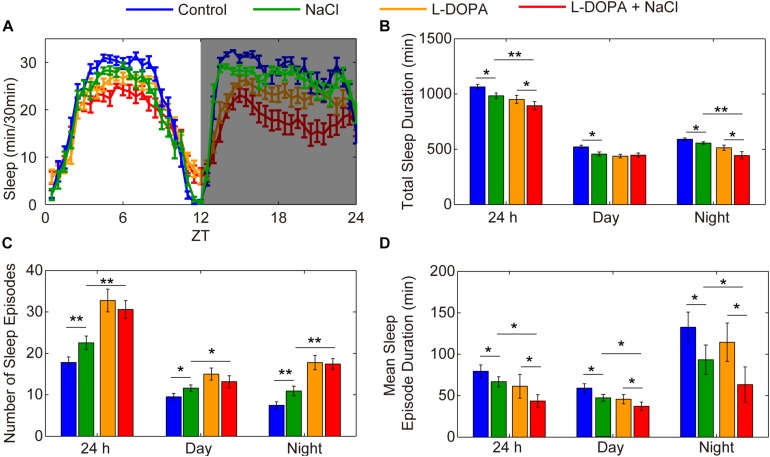
Administration of L-DOPA enhances the effects of HSD on sleep. **(A–D)** Two-to-three-day-old male flies were fed with a sucrose/agar diet, a diet supplemented with 1% NaCl, a diet supplemented with 1 mg/mL L-DOPA, or a diet supplemented with 1 mg/mL L-DOPA and 1% NaCl (*n* = 50–60 per group), and the following were determined for a 24 h period, in daytime, and in nighttime: **(A)** minutes of sleep per 30-minute period, **(B)** average sleep duration, **(C)** number of sleep episodes, and **(D)** mean sleep episode duration. Values plotted are means ± S.D.; ^∗^*P* < 0.05; ^∗∗^*P* < 0.01.

To further confirm the importance of DA neurons in regulating HSD-induced sleep changes, we explored the effect of activation of DA neurons on sleep. Under the control of *TH*-GAL4, we expressed UAS-*NaChBac*, which is a voltage-sensitive sodium channel derived from bacteria that can be used to depolarize neurons and increase their excitability (Ren et al., 2001). Night sleep was significantly decreased in the *TH*-Gal4 flies expressing UAS-*NaChBac* compared to control flies (Supplementary Figures S10A,B). Flies expressing *NaChBac* in *TH*-positive cells also showed an increase in the number of sleep episodes and a decrease in the mean sleep episode duration in both daytime and nighttime (Supplementary Figures S10C,D), which is similar to the sleep-fragmentation phenotype induced by HSD. Given that the *NaChBac* channel increases the excitability of DA cells and presumably stimulates the release of dopamine, we hypothesized that feeding the *TH*-Gal4 > UAS-*NaChBac* flies with HSD would elevate the effects. As predicted, supplementation of food with 1% sodium chloride resulted in a further decrease in total sleep duration and mean sleep episode duration for the *TH*-Gal4 > UAS-*NaChBac* flies compared to the flies given sucrose/agar food (Supplementary Figures S10B–D). However, the number of sleep episodes in *TH*-Gal4 > UAS-*NaChBac* flies with HSD was not increased compared to the flies given sucrose/agar food (Supplementary Figure S10C), which needs to be investigated further. Moreover, to exclude the possibility that HSD may increase the magnitude of neural activation via activating NaChBac and not via stimulating the release of dopamine, we also employed the strategy by using L-DOPA to target dopamine receptors. We found that flies fed with L-DOPA and 1% sodium chloride had a more exacerbated sleep-fragmentation phenotype when compared to flies only fed with L-DOPA or sodium chloride (Figure 8). However, the effect is not as strong as in the *TH*-Gal4 > UAS-*NaChBac* flies fed with sodium chloride (Supplementary Figures S10B–D), suggesting that, in addition to the increase in dopamine release, there might be an increase in the driving force for Na^+^ currents through NaChBac. Thus, we concluded that the sleep phenotype observed in the flies fed a HSD is dopaminergic system-dependent.

## Discussion

Diets that are high in salt have long been associated with high blood pressure, which raises the risk for heart disease, stroke, kidney failure, and other health problems (Farquhar et al., 2015). Studies have also linked salt intake with brain health, but the mechanisms involved are poorly understood. HSD causes changes in the guts of mice that have been seen to lead to reduced blood flow to the brain and impaired cognition (Faraco et al., 2018). The mechanism involved was independent of blood pressure. Moreover, HSD has been shown to enhance hippocampal oxidative stress in mice, resulting in cognitive impairment (Liu et al., 2014). In addition, excess salt has been reported to disrupt the blood-brain barrier (BBB) (Zhang et al., 2015). The BBB is a highly selective, semipermeable border that separates the circulating blood from the brain and extracellular fluid in the central nervous system (Daneman and Prat, 2015). In *Drosophila*, surface glia, which are the fly equivalent of the BBB, mediate the effect of dynamin, an endocytosis protein, on sleep regulation (Artiushin et al., 2018). However, whether HSD affects the function of the BBB to induce sleep changes remains to be investigated.

Aging is an extremely complex phenomenon caused by the loss of cellular homeodynamics and, consequently, the decline of physiological functions. The process of aging is affected by both genetic and environmental (e.g., diet) factors. A HSD speeds up the cellular aging process (Zhu et al., 2015). Compared with those who ate a low-salt diet, obese or overweight teenagers aged from 14 to 18 who ate a HSD had shorter telomeres, the protective caps at the end of chromosomes (Zhu et al., 2015). High levels of body fat are also known to hasten the shortening of telomeres in the elderly (Njajou et al., 2012). This study suggests that HSD and obesity may act synergistically to accelerate cellular aging (Zhu et al., 2015). Moreover, human sleep problems, particularly decrease in the amount of quality sleep or deep sleep, become more frequent with aging (Dijk et al., 2010). Here, we showed that HSD induced sleep fragmentation and reduced sleep maintenance in young flies, a pattern that is similar to that observed in older individuals. This provides a new link between HSD and aging. However, we found that a HSD is mediated by increased dopamine release, while previous studies have found a loss of dopamine production in aged flies (Neckameyer et al., 2000). High salt alters reactive oxygen species (Li et al., 2018) and mitochondrial function (Stocher et al., 2018), and these are both related with the aging process (DiLoreto and Murphy, 2015). It remains to be investigated whether and how HSD affects aging-related processes to cause sleep disruption in young individuals.

In this study, we found that HSD-induced sleep changes were both circadian rhythm-dependent and dopaminergic system-dependent. This is not surprising, because a link between circadian rhythm and dopaminergic systems has been reported. Specifically, in mammals, genetic deletion of the circadian nuclear receptor *Rev-erbα* increased midbrain dopaminergic tone at dusk (Kim et al., 2017). REV-ERBα antagonizes NURR1-induced activation of the *TH* promoter via binding to the ROR/REV-ERB and NGFI-B response elements, thereby contributing to the circadian rhythmicity of the dopaminergic system (Kim et al., 2017). Moreover, rhythms in clock gene expression in the dorsal striatum are sensitive to changes in dopamine release (Verwey et al., 2016). In *Drosophila*, there is a circadian-independent requirement for *Clk* in brain circadian neurons to maintain the PPL1 subset of dopaminergic neurons (Vaccaro et al., 2017). Here, we found that HSD elevates TH levels in the dopaminergic neurons. This is consistent with what has been previously reported in studies of mammals: HSD increases both dopamine levels and DA/L-DOPA ratios in the renal cortex of adult rats (Vieira-Coelho et al., 1999). In addition, the observed increase in urinary dopamine excretion in rats fed HSD is mainly caused by the enhancement of extraneural dopamine production by the kidney (Hayashi et al., 1991). Moreover, HSD markedly increases the tissue levels of dopamine (Lucas-Teixeira et al., 2000). Dopamine D2 receptors are involved in sodium handling and blood pressure control (Ueda et al., 2003), and D3 receptor deficiency can underlie salt-dependent hypertension (Luippold et al., 2001). It has been shown that stimulating dopamine neurons in the midbrain reduces salt intake (Sandhu et al., 2018), suggesting that HSD-induced dopaminergic system dysfunction may be a stress response.

There is a complex relationship between diet and sleep. Sleep duration and quality have an impact on food consumption and choice in both adults and children (St-Onge et al., 2016; Frank et al., 2017). Studies have also shown that an individual’s dietary patterns can affect their sleep (St-Onge et al., 2016; Frank et al., 2017). For instance, a high-carbohydrate plus low-fat diet is associated with poorer sleep quality (Frank et al., 2017), and protein and carbohydrate deficiencies are associated with shorter sleep duration (St-Onge et al., 2016). Dietary quality and the intake of specific nutrients can affect hormonal signaling pathways to alter sleep duration and quality (Frank et al., 2017). For example, tryptophan is an amino acid precursor to the sleep-regulating hormone serotonin, and natural D-serine is an effective co-agonist of the *N*-methyl-D-aspartate subtype of glutamate receptor, which is involved in sleep regulation (Dai et al., 2019). There is also evidence linking HSD with increased levels of the hormone cortisol, and these excess levels may affect sleep (Baudrand et al., 2014).

Poor sleep quality can have long-term consequences. It increases the risk of many chronic diseases and decreases quality of life, with financial burdens on the economy (Frank et al., 2017). Given the increasing prevalence of reduced sleep quality and its costs, identification of dietary factors that influence sleep either positively or negatively will improve public health. Here, we showed that a HSD reduces sleep duration and that flies fed a HSD awaken more often during sleep than flies fed a standard diet. Our experiments indicate that the circadian and dopaminergic systems mediate the effects of a HSD.

## Data Availability Statement

The raw data supporting the conclusions of this manuscript will be made available by the authors, without undue reservation, to any qualified researcher.

## Author Contributions

WC conceived the project and wrote the original draft. WC and YY designed the experiments. JX, DW, SL, and WC performed the experiments. WC, JX, and SL performed the analysis. WC, GY, and YY reviewed and edited the manuscript.

## Conflict of Interest

The authors declare that the research was conducted in the absence of any commercial or financial relationships that could be construed as a potential conflict of interest.

## Footnotes

1 https://www.flyrnai.org/flyprimerbank

## Supplementary Material

FIGURE S1 HSD causes fragmented sleep in the young female flies. **(A–D)** Two-to-three-day-old female flies (*n* = 48–64 per group) were fed a sucrose/agar diet (blue), 0.05% NaCl (yellow), 0.25% NaCl (green), or 1% NaCl (red), and the following were determined over a 24 h period, in daytime, and in nighttime: **(A)** minutes of sleep per 30-minute period average, **(B)** sleep duration, **(C)** number of sleep episodes, and **(D)** mean duration of each sleep episode. Values plotted are means ± S.D.; ^∗∗^*P* < 0.01 relative to sucrose/agar diet control.

FIGURE S2 Waking activity is normal in young male and female flies fed with various concentrations of NaCl. Two-to-three-day-old male (left) and female (right) flies (*n* = 48–64 per group) were fed a sucrose/agar diet (blue), 0.05% NaCl (yellow), 0.25% NaCl (green), or 1% NaCl (red), and activity while awake was calculated as the total beam breaks divided by the number of minutes with activity. Values plotted are means ± S.D. Differences from sucrose/agar diet control are not significant.

FIGURE S3 Sleep fragmentation effects of HSD are reversible by returning dietary sodium intake to normal. Two-to-three-day-old male flies (*n* = 39–48 per group) were fed 1% NaCl or normal food following a food transfer on the 5th day. After 4 days of normal food or HSD sleep assays, flies were transferred to the opposite foods on the 5th day. Sleep episodes were determined over a 24 h period, in daytime, and in nighttime. **(A)** Number of sleep episodes. **(B)** Mean duration of each sleep episode. Values plotted are means ± S.D.

FIGURE S4 Effects of potassium chloride or calcium chloride on sleep. **(A)** Effect of potassium chloride on sleep. Two-to-three-day-old male flies (*n* = 32–48 per group) were fed a sucrose/agar diet, 0.05, 0.25, or 1% KCl, and the sleep parameters were determined over a 24 h period, in daytime, and in nighttime. **(B)** Effect of calcium chloride on sleep. Two-to-three-day-old male flies (*n* = 35–50 per group) were fed a sucrose/agar diet, 0.05, 0.25, or 1% CaCl_2_, and the sleep parameters were determined over a 24 h period, in daytime, and in nighttime. ^∗^*P* < 0.05; ^∗∗^*P* < 0.01.

FIGURE S5 HSD shortens the lifespan of young flies in a dose-dependent manner. Young or 50-day-old male flies (*n* = 50 per group) were fed a diet supplemented or not with various concentrations of NaCl. Percent survival versus time is plotted. The experiment was repeated twice. A log-rank (mantel-Cox) test showed that the survival curves are significantly different. ^****^*P* < 0.0001.

FIGURE S6 HSD causes fragmented sleep in young *ry*^506^ male flies. **(A–D)** Two-to-three-day-old male flies (*n* = 32 per group) were fed a sucrose/agar diet (blue), 0.05% NaCl (yellow), 0.25% NaCl (green), or 1% NaCl (red) and the following were determined over a 24 h period, in daytime, and in nighttime: **(A)** minutes of sleep per 30-minute period average, **(B)** sleep duration, **(C)** number of sleep episodes, and **(D)** mean duration of each sleep episode. Values plotted are means ± S.D.; ^∗^*P* < 0.05; ^∗∗^*P* < 0.01 relative to sucrose/agar diet control.

FIGURE S7 HSD does not affect TH levels in PAM neurons. **(A)** Representative control whole brain stained with anti-TH. The location of the PAM neurons is indicated. Scale bars: 100 μm. **(B)** Representative images of PAM regions of control and HSD flies stained with anti-TH. Scale bars: 10 μm. **(C)** Quantification of TH intensity in the PAM neurons (*n* = 8–10 per group). Values plotted are means ± S.D.; n.s. means not significant relative to levels in the control.

FIGURE S8 TH protein levels are abnormally low in the brains of *TH*-KO^Gal4/+^ flies. **(A)** Representative whole brains of wild-type and *TH*-KO^Gal4/+^ flies stained with anti-TH. PPL1, PPL2, PPM3, PPM12, and PAM neuron regions are indicated. Scale bars: 100 μm. **(B)** Quantitative analysis of the intensity of anti-TH staining in brains of control and *TH*-KO^Gal4/+^ flies (*n* = 8 per group). Values plotted are means ± S.D.; ^****^*P* < 0.0001 relative to control.

FIGURE S9 Sleep behaviors of DA-silencing flies at permissive temperature. **(A–D)** *TH*-Gal4 > UAS-*shi*^ts^ flies and control flies (*n* = 35–40 per group) were reared at 18°C and were then transferred to 22°C and fed a sucrose/agar diet or a diet supplemented with 1% NaCl. The following were determined for a 24 h period, in daytime, and in nighttime: **(A)** minutes of sleep per 30-minute period, **(B)** average sleep duration, **(C)** number of sleep episodes, and **(D)** mean sleep episode duration. Values plotted are means ± S.D.; ^∗^*P* < 0.05; ^∗∗^*P* < 0.01.

FIGURE S10 Activation of DA neurons enhances the effects of HSD on sleep. **(A–D)** *TH*-Gal4 > UAS-*NaChBac* DA neuron-activating and control flies were fed with or without 1% NaCl (*n* = 35–45 per group), and the following were determined for a 24 h period, in daytime, and in nighttime: **(A)** minutes of sleep per 30-minute period, **(B)** average sleep duration, **(C)** number of sleep episodes, and **(D)** mean sleep episode duration. Values plotted are means ± S.D.; ^∗∗^*P* < 0.01; ^∗∗∗^*P* < 0.001.
