# Supplementary figures and images for: High-Salt Diet Causes Sleep Fragmentation in Young Drosophila Through Circadian Rhythm and Dopaminergic Systems
Source: Front Neurosci. 2019 Nov 29;13:1271. doi: 10.3389/fnins.2019.01271 (PMC6895215; doi:10.3389/fnins.2019.01271)

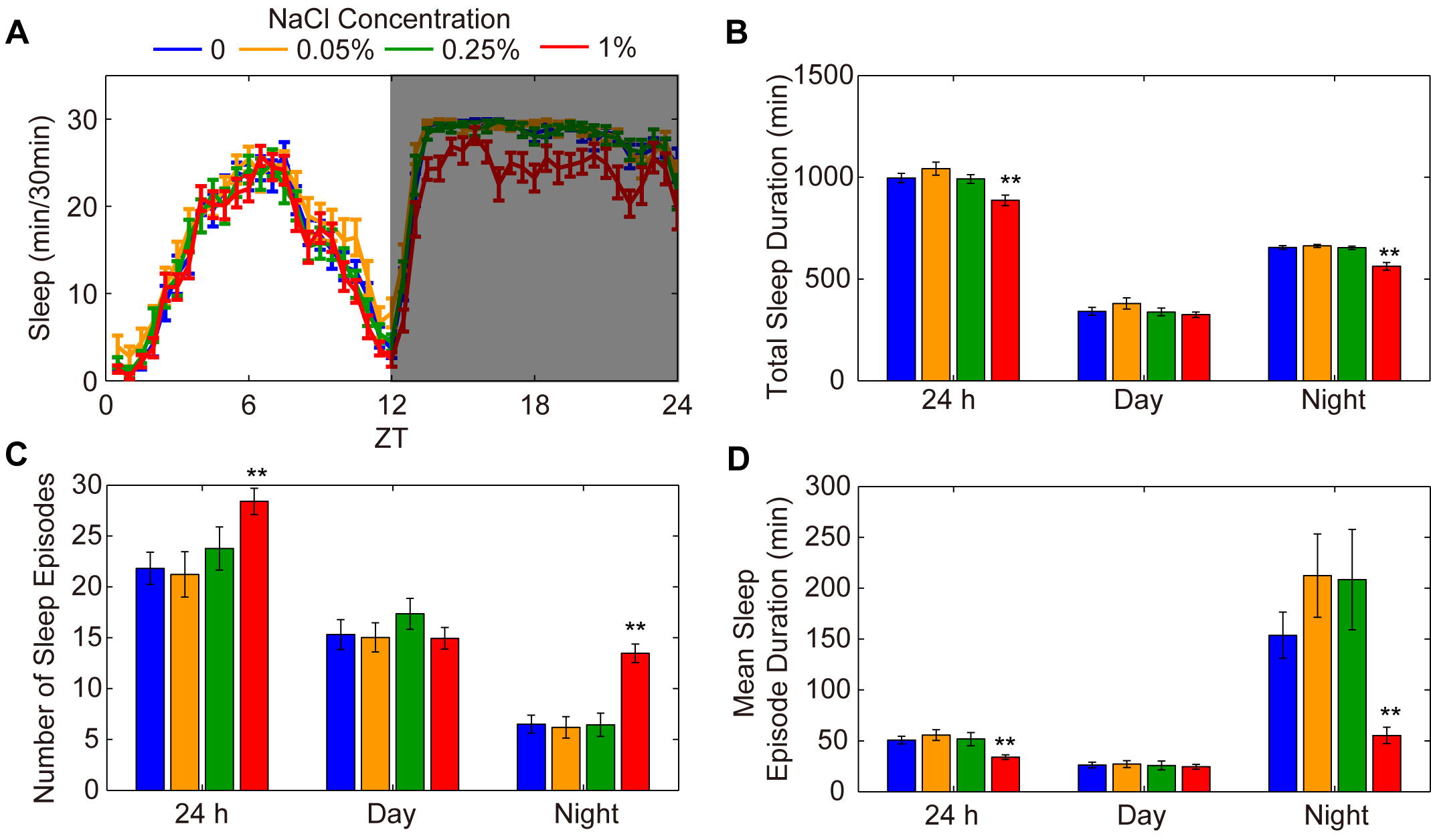

Supplement: FIGURE S1 — HSD causes fragmented sleep in the young female flies. (A–D) Two-to-three-day-old female flies (n = 48–64 per group) were fed a sucrose/agar diet (blue), 0.05% NaCl (yellow), 0.25% NaCl (green), or 1% NaCl (red), and the following were determined over a 24 h period, in daytime, and in nighttime: (A) minutes of sleep per 30-minute period average, (B) sleep duration, (C) number of sleep episodes, and (D) mean duration of each sleep episode. Values plotted are means ± S.D.; ∗∗P < 0.01 relative to sucrose/agar diet control. [file Image_1.TIF]

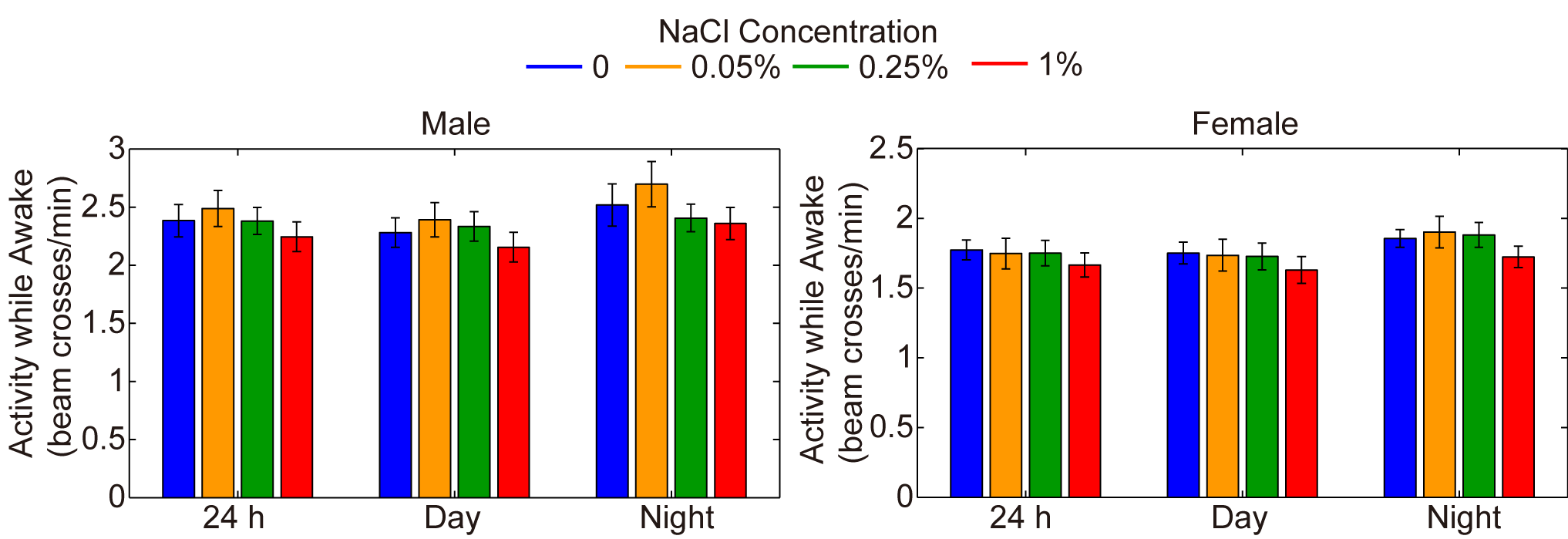

Supplement: FIGURE S2 — Waking activity is normal in young male and female flies fed with various concentrations of NaCl. Two-to-three-day-old male (left) and female (right) flies (n = 48–64 per group) were fed a sucrose/agar diet (blue), 0.05% NaCl (yellow), 0.25% NaCl (green), or 1% NaCl (red), and activity while awake was calculated as the total beam breaks divided by the number of minutes with activity. Values plotted are means ± S.D. Differences from sucrose/agar diet control are not significant. [file Image_2.TIF]

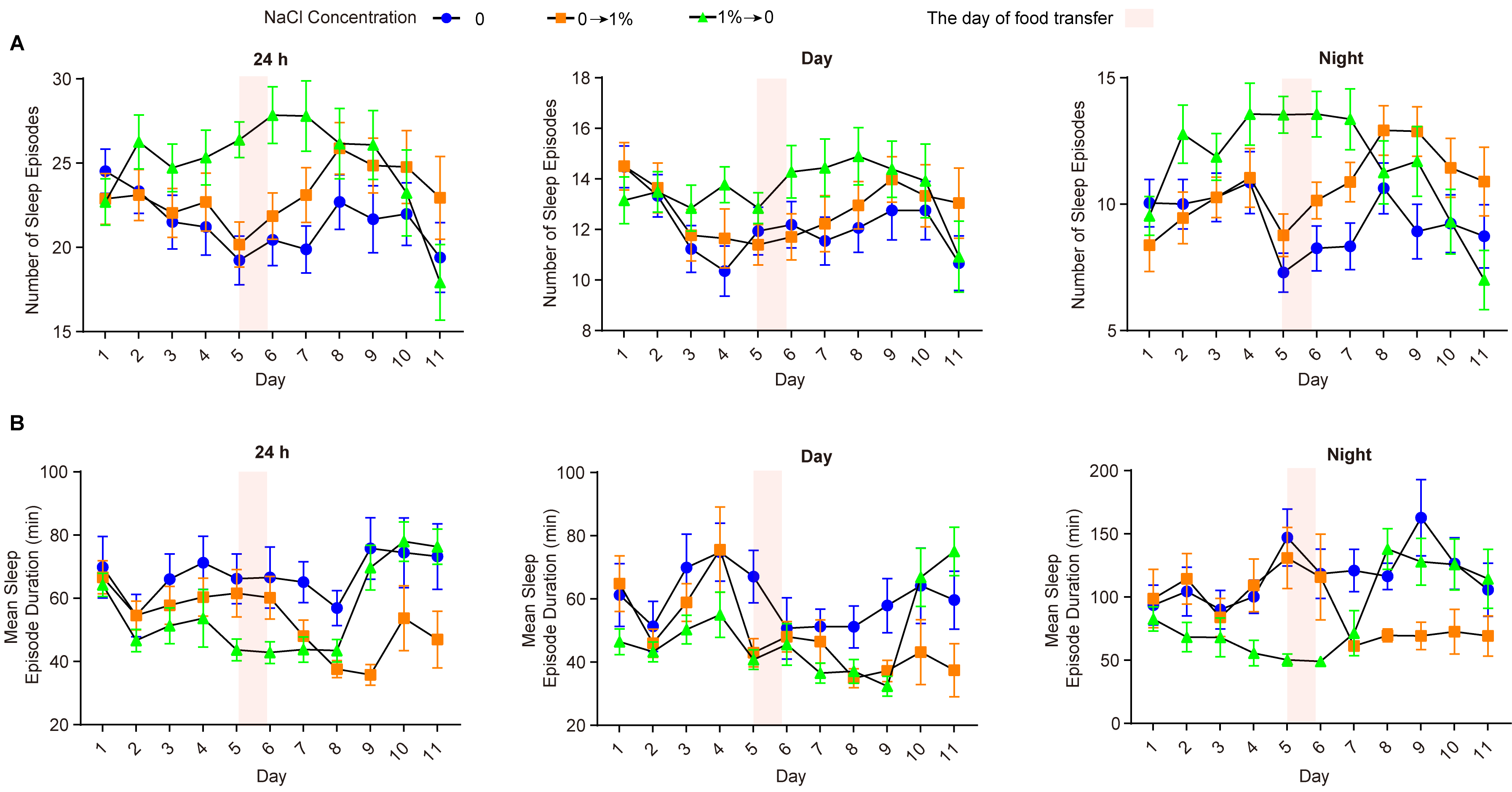

Supplement: FIGURE S3 — Sleep fragmentation effects of HSD are reversible by returning dietary sodium intake to normal. Two-to-three-day-old male flies (n = 39–48 per group) were fed 1% NaCl or normal food following a food transfer on the 5th day. After 4 days of normal food or HSD sleep assays, flies were transferred to the opposite foods on the 5th day. Sleep episodes were determined over a 24 h period, in daytime, and in nighttime. (A) Number of sleep episodes. (B) Mean duration of each sleep episode. Values plotted are means ± S.D. [file Image_3.TIF]

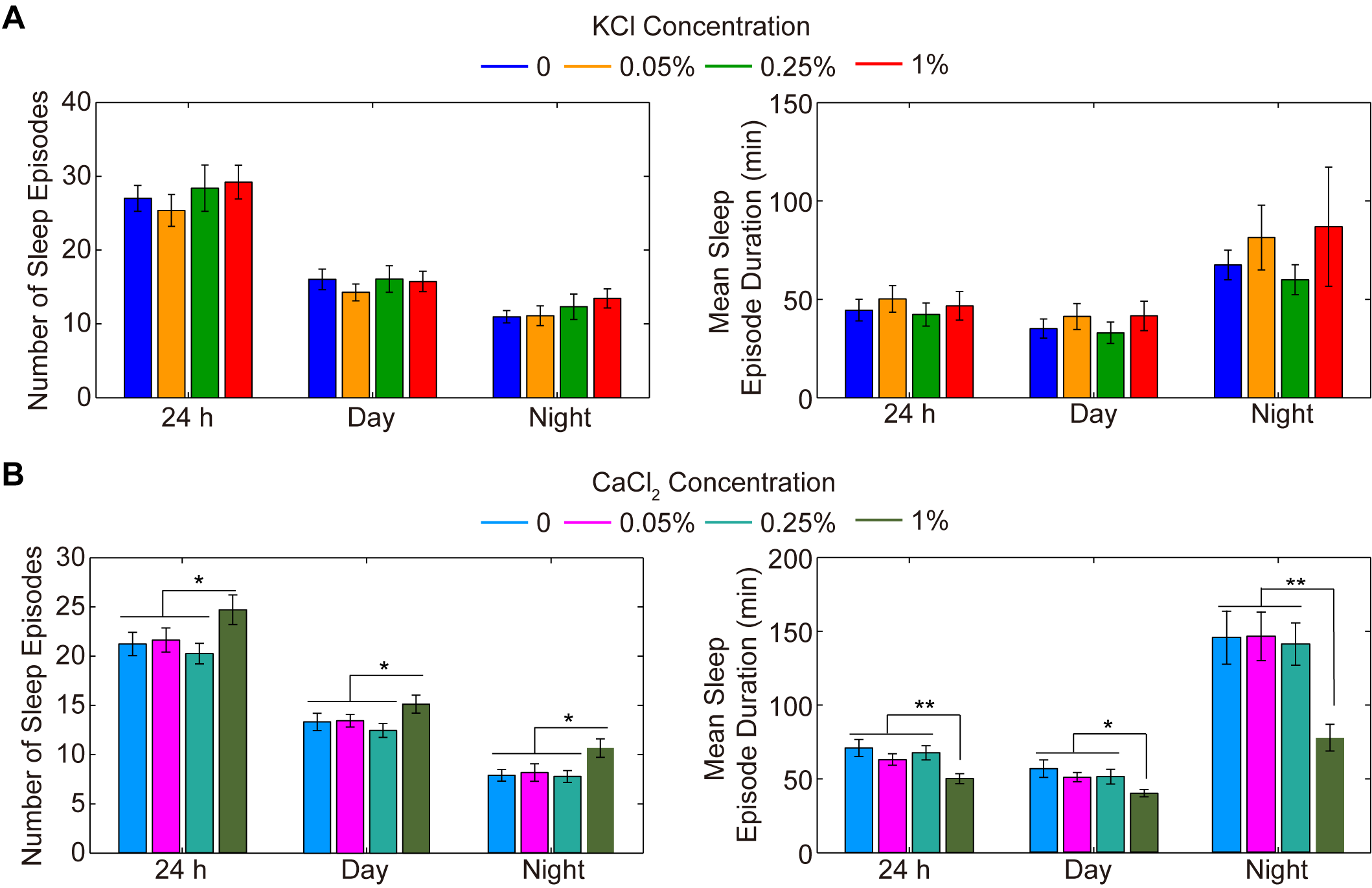

Supplement: FIGURE S4 — Effects of potassium chloride or calcium chloride on sleep. (A) Effect of potassium chloride on sleep. Two-to-three-day-old male flies (n = 32–48 per group) were fed a sucrose/agar diet, 0.05, 0.25, or 1% KCl, and the sleep parameters were determined over a 24 h period, in daytime, and in nighttime. (B) Effect of calcium chloride on sleep. Two-to-three-day-old male flies (n = 35–50 per group) were fed a sucrose/agar diet, 0.05, 0.25, or 1% CaCl2, and the sleep parameters were determined over a 24 h period, in daytime, and in nighttime. ∗P < 0.05; ∗∗P < 0.01. [file Image_4.TIF]

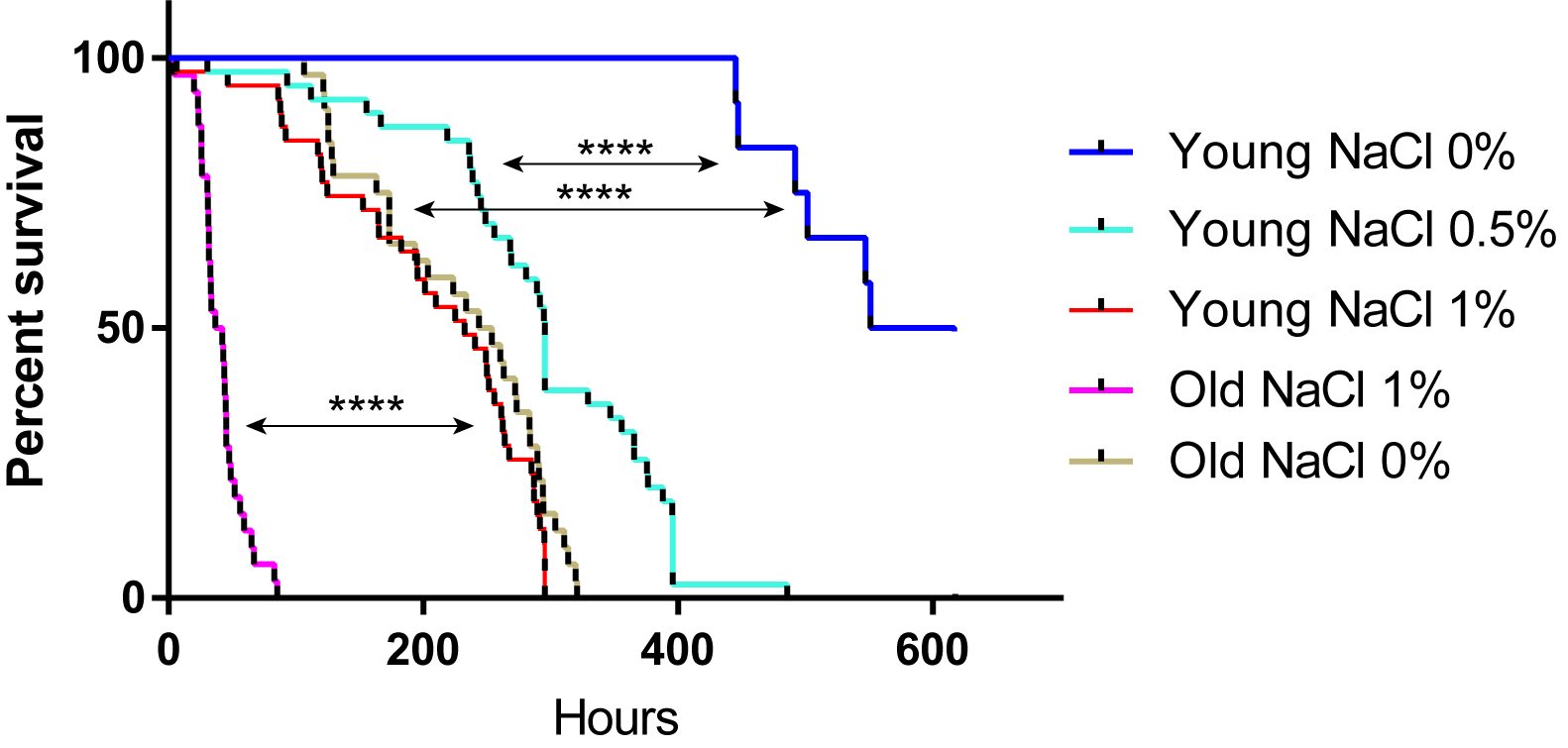

Supplement: FIGURE S5 — HSD shortens the lifespan of young flies in a dose-dependent manner. Young or 50-day-old male flies (n = 50 per group) were fed a diet supplemented or not with various concentrations of NaCl. Percent survival versus time is plotted. The experiment was repeated twice. A log-rank (mantel-Cox) test showed that the survival curves are significantly different. ****P < 0.0001. [file Image_5.tif]

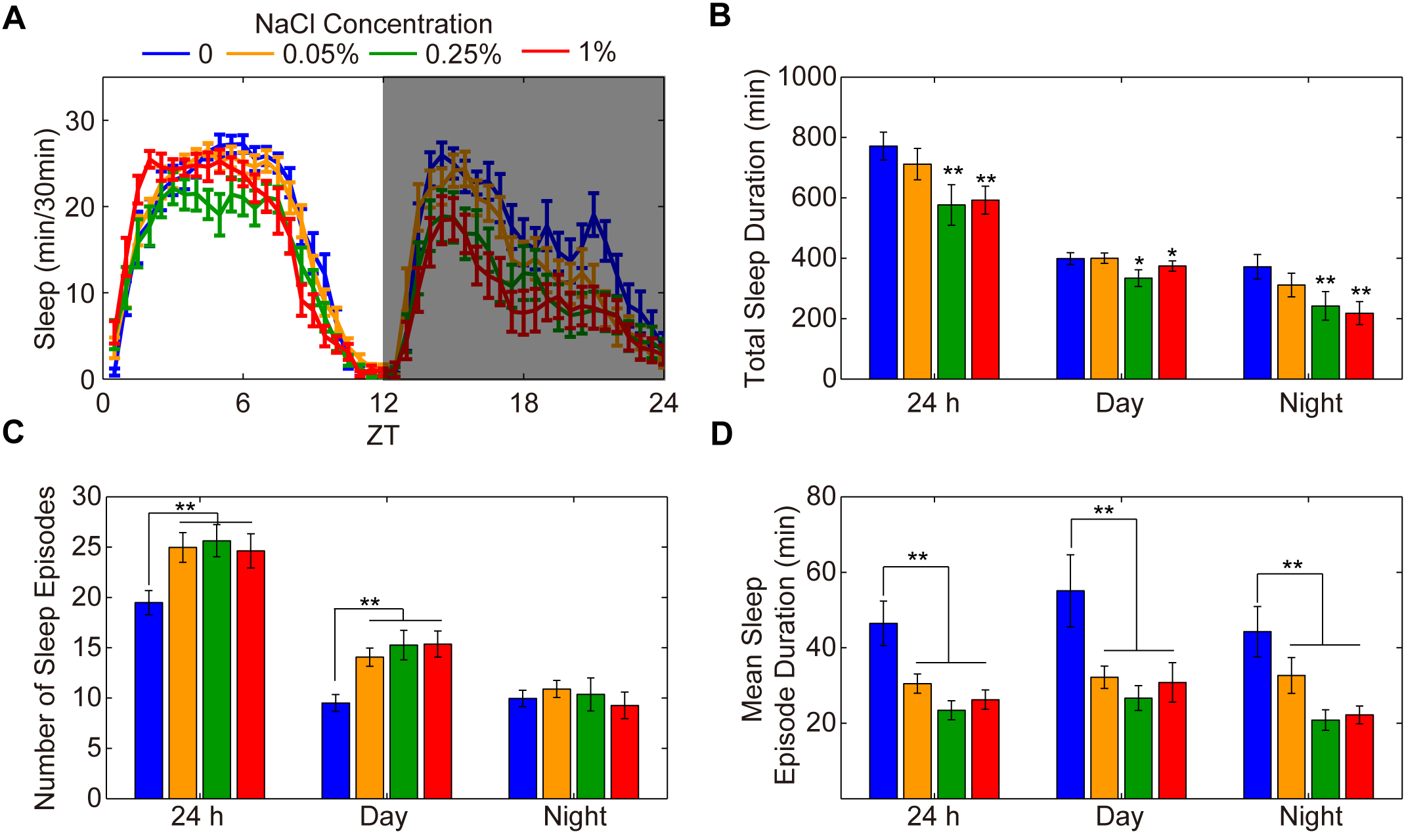

Supplement: FIGURE S6 — HSD causes fragmented sleep in young ry506 male flies. (A–D) Two-to-three-day-old male flies (n = 32 per group) were fed a sucrose/agar diet (blue), 0.05% NaCl (yellow), 0.25% NaCl (green), or 1% NaCl (red) and the following were determined over a 24 h period, in daytime, and in nighttime: (A) minutes of sleep per 30-minute period average, (B) sleep duration, (C) number of sleep episodes, and (D) mean duration of each sleep episode. Values plotted are means ± S.D.; ∗P < 0.05; ∗∗P < 0.01 relative to sucrose/agar diet control. [file Image_6.TIF]

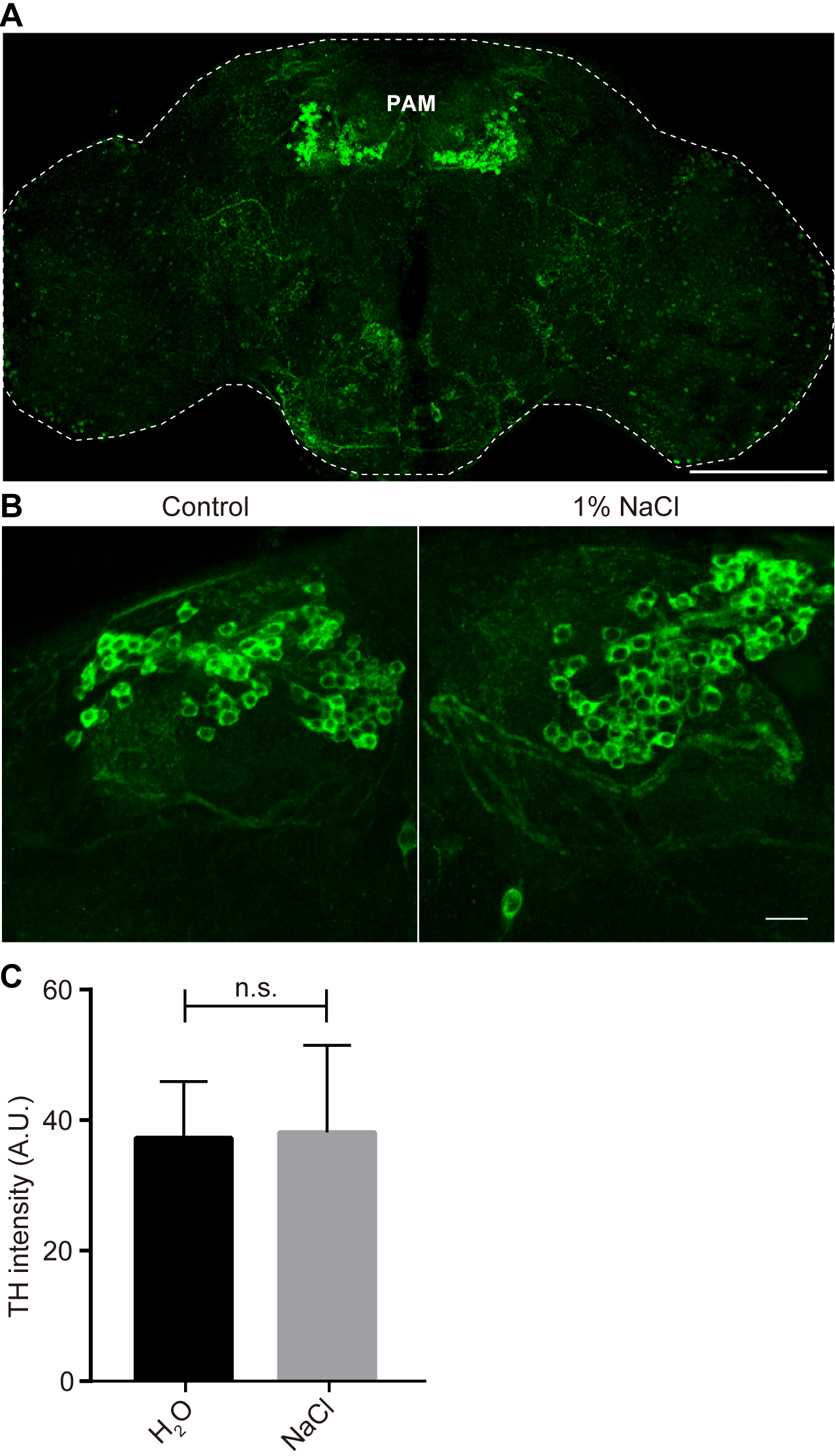

Supplement: FIGURE S7 — HSD does not affect TH levels in PAM neurons. (A) Representative control whole brain stained with anti-TH. The location of the PAM neurons is indicated. Scale bars: 100 μm. (B) Representative images of PAM regions of control and HSD flies stained with anti-TH. Scale bars: 10 μm. (C) Quantification of TH intensity in the PAM neurons (n = 8–10 per group). Values plotted are means ± S.D.; n.s. means not significant relative to levels in the control. [file Image_7.TIF]

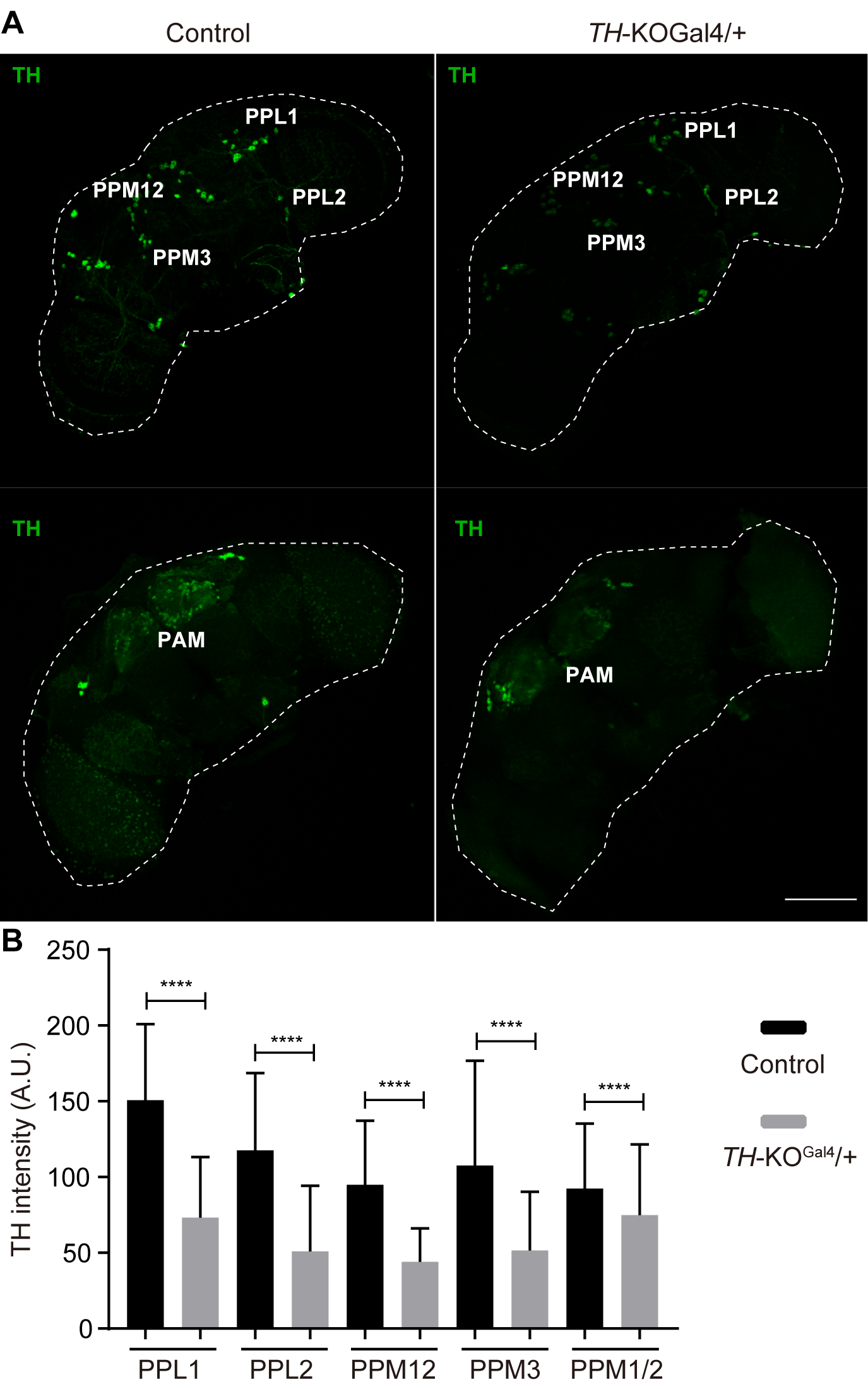

Supplement: FIGURE S8 — TH protein levels are abnormally low in the brains of TH-KOGal4/+ flies. (A) Representative whole brains of wild-type and TH-KOGal4/+ flies stained with anti-TH. PPL1, PPL2, PPM3, PPM12, and PAM neuron regions are indicated. Scale bars: 100 μm. (B) Quantitative analysis of the intensity of anti-TH staining in brains of control and TH-KOGal4/+ flies (n = 8 per group). Values plotted are means ± S.D.; ****P < 0.0001 relative to control. [file Image_8.TIF]

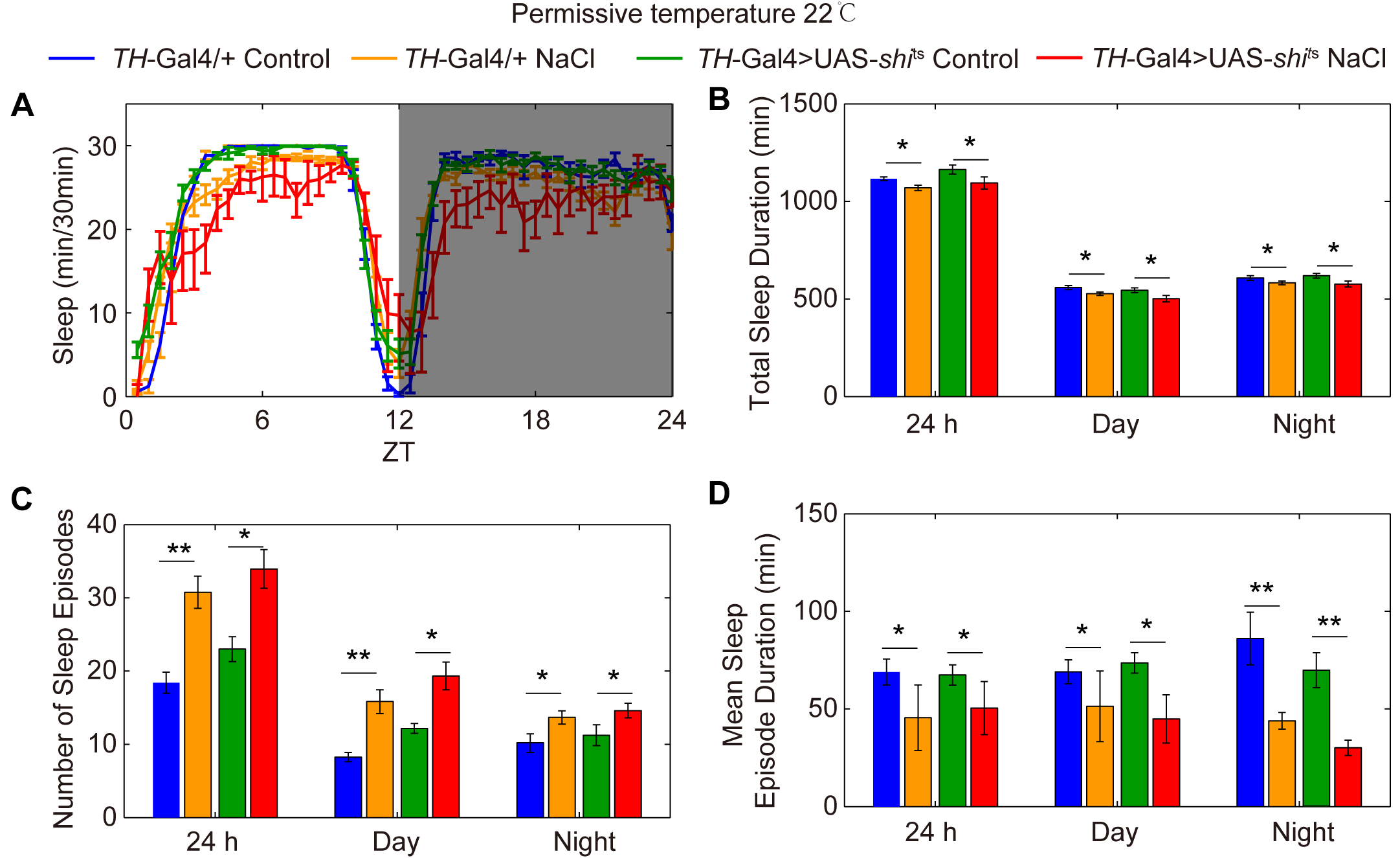

Supplement: FIGURE S9 — Sleep behaviors of DA-silencing flies at permissive temperature. (A–D) TH-Gal4 > UAS-shits flies and control flies (n = 35–40 per group) were reared at 18°C and were then transferred to 22°C and fed a sucrose/agar diet or a diet supplemented with 1% NaCl. The following were determined for a 24 h period, in daytime, and in nighttime: (A) minutes of sleep per 30-minute period, (B) average sleep duration, (C) number of sleep episodes, and (D) mean sleep episode duration. Values plotted are means ± S.D.; ∗P < 0.05; ∗∗P < 0.01. [file Image_9.TIF]

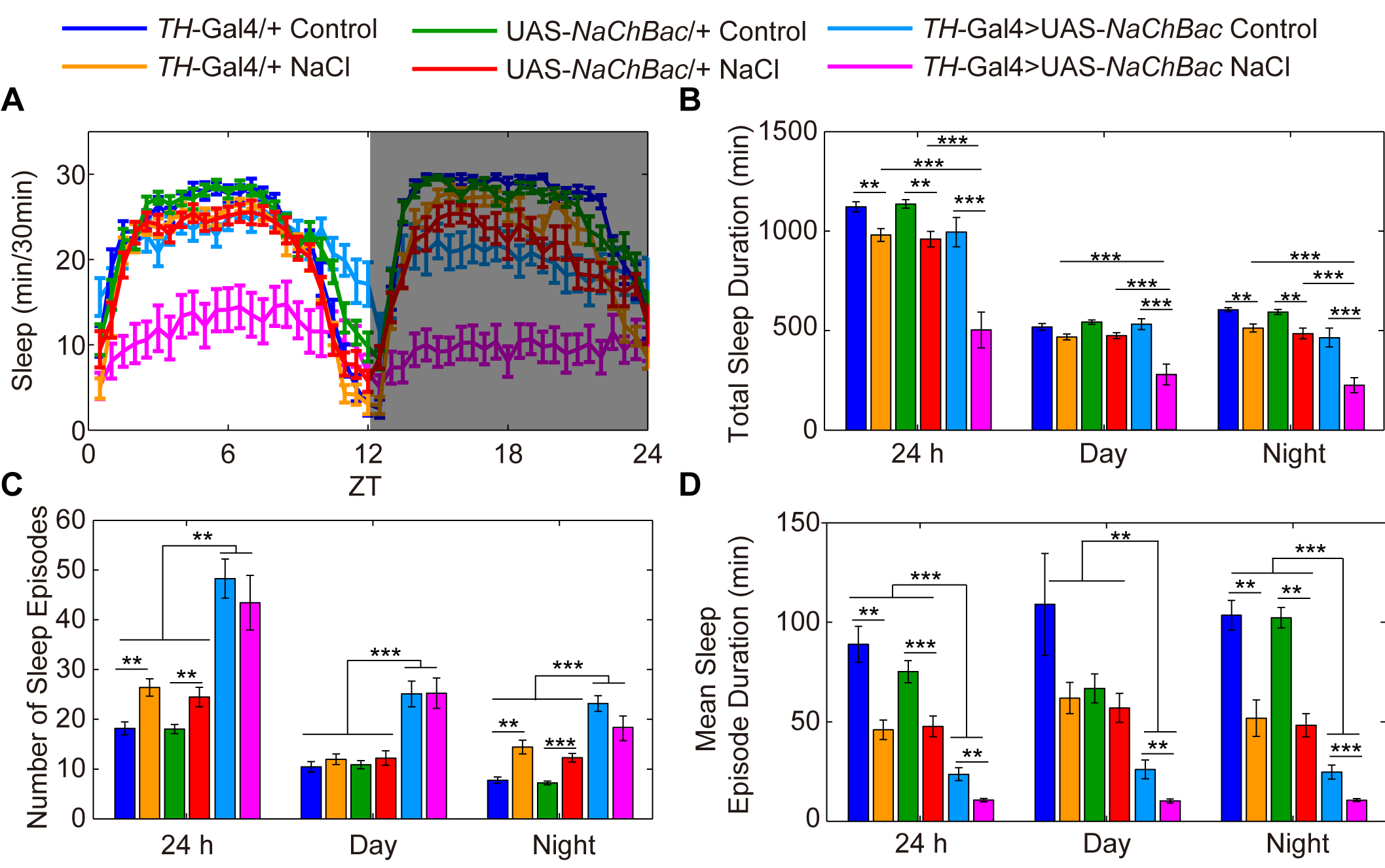

Supplement: FIGURE S10 — Activation of DA neurons enhances the effects of HSD on sleep. (A–D) TH-Gal4 > UAS-NaChBac DA neuron-activating and control flies were fed with or without 1% NaCl (n = 35–45 per group), and the following were determined for a 24 h period, in daytime, and in nighttime: (A) minutes of sleep per 30-minute period, (B) average sleep duration, (C) number of sleep episodes, and (D) mean sleep episode duration. Values plotted are means ± S.D.; ∗∗P < 0.01; ∗∗∗P < 0.001. [file Image_10.TIF]
